# Supplementary material for: A Critical View of the Application of the APEX Software (Aqueous Photochemistry of Environmentally-Occurring Xenobiotics) to Predict Photoreaction Kinetics in Surface Freshwaters
Source: Molecules. 2019 Dec 18;25(1):9. doi: 10.3390/molecules25010009 (PMC7017383; doi:10.3390/molecules25010009)
Supplement: Supplementary file 1 [file molecules-25-00009-s001.zip › 3.2.4_gcc-4.4.0.zip!/3.2.4_gcc-4.4.0/3.2.4_gcc-4.4.0/doc/octave/html/interpreter/Input-and-Output.html]

Input and Output - Untitled


Next: Plotting,
Previous: Debugging,
Up: Top


---

## 14 Input and Output

Octave supports several ways of reading and writing data to or from the
prompt or a file. The simplest functions for data Input and Output
(I/O) are easy to use, but only provides limited control of how
data is processed. For more control, a set of functions modelled
after the C standard library are also provided by Octave.

- Basic Input and Output- C-Style I/O Functions
